# 

**Published:** 1887-07-23

**Authors:** 


PRICE ONE PENNY.

No. 43, Vol. II.

July 23rd, 1887.

[Registered at the General Post Office as a Newspaper.]

Per Annum, 6s. 6d., post free.

Monthly Parts, 6d.; post free, 7½d.

Ditto per Ann., 7s. 6d., post free.

### THE HOSPITAL

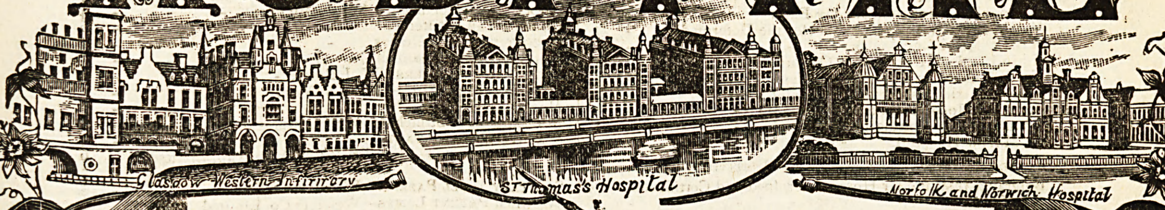

An Institution, Family, and Parochial Journal of

Hospitals, Asylums, & all Agencies for the Care of the Sick, Criticism & News.

